# Establishment of multiplex RT-PCR to detect fusion genes for the diagnosis of Ewing sarcoma

**DOI:** 10.1186/s13000-021-01164-6

**Published:** 2021-11-08

**Authors:** Hitomi Ueno-Yokohata, Hajime Okita, Keiko Nakasato, Chikako Kiyotani, Motohiro Kato, Kimikazu Matsumoto, Nobutaka Kiyokawa, Atsuko Nakazawa, Takako Yoshioka

**Affiliations:** 1 Department of Pediatric Hematology and Oncology Research, National Research Institute for Child Health and Development, Tokyo, 157-8535 Japan; 2 Division of Diagnostic Pathology, Keio University School of Medicine, Tokyo, 160-8582 Japan; 3 Department of Pathology, Keio University School of Medicine, Tokyo, 160-8582 Japan; 4 Department of Pathology, National Center for Child Health and Development, Tokyo, 157-8535 Japan; 5 Children’s Cancer Center, National Center for Child Health and Development, Tokyo, 157-8535 Japan; 6 Department of Pediatrics, Graduate School of Medicine, University of Tokyo, Tokyo, 113-8655 Japan; 7 Department of Clinical Research, Saitama Children’s Medical Center, Saitama, Saitama 330-8777 Japan

**Keywords:** Ewing sarcoma, Multiplex RT–PCR, Genetic diagnosis, Fusion gene, *EWSR1*, Transcription factor, Breakpoint

## Abstract

**Background:**

Detection of the tumor-specific *EWSR1/FUS-ETS* fusion gene is essential to diagnose Ewing sarcoma. Reverse transcription–polymerase chain reaction (RT–PCR) and fluorescence in situ hybridization are commonly used to detect the fusion gene, and assays using next-generation sequencing have recently been reported. However, at least 28 fusion transcript variants have been reported, making rapid and accurate detection difficult.

**Methods:**

We constructed two sets of multiplex PCR assays and evaluated their utility using cell lines and clinical samples.

**Results:**

*EWSR1/FUS-ETS* was detected in five of six tumors by the first set, and in all six tumors by the second set. The fusion gene detected only by the latter was *EWSR1-ERG*, which completely lacked exon 7 of *EWSR1*. The fusion had a short N-terminal region of *EWSR1* and showed pathologically atypical features.

**Conclusions:**

We developed multiplex RT–PCR assays to detect *EWSR1-ETS* and *FUS-ETS* simultaneously. These assays will aid the rapid and accurate diagnosis of Ewing sarcoma. In addition, variants of *EWSR1/FUS-ETS* with a short N-terminal region that may have been previously missed can be easily detected.

**Supplementary Information:**

The online version contains supplementary material available at 10.1186/s13000-021-01164-6.

## Background

Ewing sarcoma primarily occurs in the bones and soft tissues of children and young adults. It is characterized by fusion genes between a gene of the RNA-binding FET family (*EWSR1* or *FUS*) with a gene of the ETS-transcription factor family (*FLI1*, *ERG*, *ETV1*, *ETV4* (*E1AF*), and *FEV*) [1–5], which are called *EWSR1/FUS-ETS* fusion genes. *EWSR1-FLI1*, generated by t(11;22)(q24.3;q12.2), occurs most frequently, followed by *EWSR1-ERG*, which is generated by t(21;22)(q22.2;q12.2) [6]. Similarly, *EWSR1-ETV1*, *EWSR1-ETV4*, *EWSR1-FEV*, *FUS-ERG*, and *FUS-FEV* are rarely formed in Ewing sarcoma, and these are generated by t(7;22)(p21.2;q12.2), t(17;22)(q21.31;q12.2), t(2;22)(q35;q12.2), t(16;21)(p11.2;q22.2) and t(2;16)(q35;p11.2), respectively [7, 8]. Additionally, various exon combinations exist in *EWSR1/FUS-ETS* fusion genes. In *EWSR1-FLI1*, the combination of *EWSR1* exon 7 and *FLI1* exon 6 occurs most commonly, followed by the combination of *EWSR1* exon 7 and *FLI1* exon 5. *EWSR1* exon 7 and *ERG* exon 7 or *EWSR1* exon 7 and *ERG* exon 9 are common exon combinations in *EWSR1-ERG*. These fusion genes contain the ETS consensus sequence in-frame [9, 10]. The conserved ETS consensus sequence recognizes the ETS motif, competes with wild type ETS-transcription factors, and consequently contributes to Ewing tumorigenesis [11].

Ewing sarcoma is composed of dense and diffuse proliferation of small round blue cells with fine chromatin [12]. Generally, it lacks immunohistochemical evidence of differentiation lineages, such as muscle, bone, cartilage, fibroblast and endothelium. Diffuse membranous CD99 immunoreactivity is a hallmark of this tumor and more than 90% of tumors were reported to have *EWSR1/FUS-ETS*. Therefore, detection of the fusion gene is important to diagnose Ewing sarcoma. Fluorescence in situ hybridization (FISH), reverse transcription-polymerase chain reaction (RT–PCR), including multiplexed assay, and targeted next-generation sequencing have been reported. FISH using an *EWSR1* or a *FUS* break-apart probe is commonly used in clinical settings, but FISH using formalin-fixed paraffin-embedded tissue may sometimes be challenging [13]. Additionally, fusion partners cannot be determined by a single break-apart assay. Many tumors, such as desmoplastic small round cell tumor, myxoid liposarcoma, clear cell sarcoma of tendons and aponeuroses, angiomatoid fibrous histiocytoma, and myoepithelioma, have a fusion gene related to *EWSR1* and *FUS* [14–18]. Therefore, we believe that the determination of the fusion partner gene is important for the differential diagnosis. RT–PCR detection is sensitive and specific, and it can determine the fusion partner. However, assays for seven different fusion genes are needed for Ewing sarcoma. Multiplex RT-PCR assay is an efficient technique. Nevertheless, multiplex RT-PCR assays for *EWSR1-ETS* have been reported, those for both *EWSR1-ETS* and *FUS-ETS* fusion genes have not [19–22]. Next-generation sequencing is a robust technique, but it is too expensive. Therefore, we aimed to create a multiplex RT–PCR system that can simultaneously detect known *EWSR1/FUS-ETS* fusion genes. Moreover, we confirmed the utility using clinical samples and plasmids.

## Materials and methods

### Clinical samples

The pathological diagnosis was confirmed by H.O., A.N., and/or T.Y. based on morphological observations and existing RT–PCR and/or FISH analysis. The clinical samples other than tumor 4 used in this study had already been identified for fusion variants by existing RT–PCR. Furthermore, the multiplex RT–PCR and sequencing analysis were performed as blind for experimenter, and the result was collated with that of existing method. Immunostaining was performed using HISTOSTAINER (NICHIREI BIOSCIENCES, Tokyo, Japan) or the BOND-III automated stainer (Leica Biosystems, Nussloch, Germany). Detailed information about the antibodies used in this study is listed in Supplementary Table S1.

### RT–PCR

**Fig. 1:**
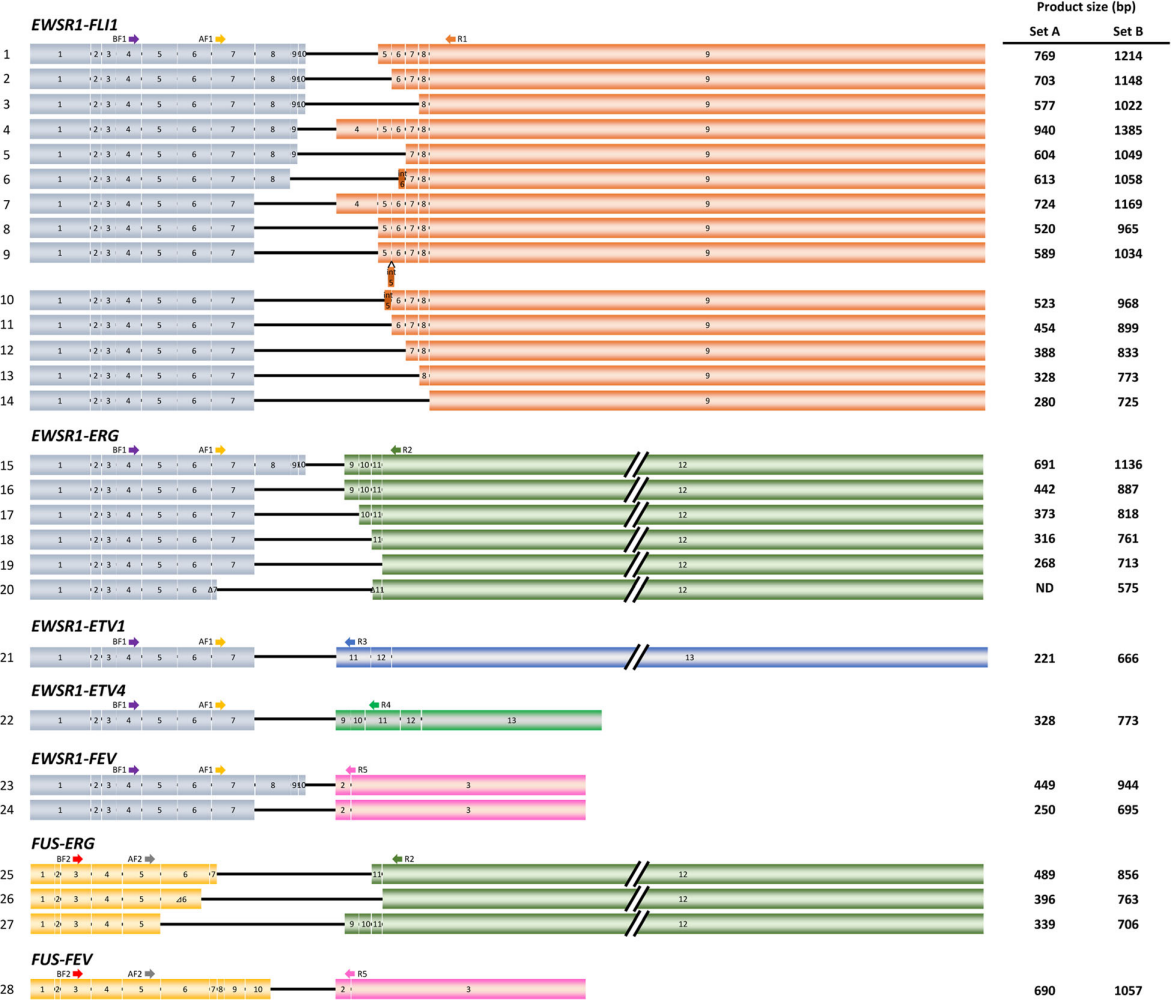
Fusion transcript variants of *EWS/FUS-ETS*. Previously described fusion transcripts are indicated. The arrows indicate the primer position listed in Table 1. ND represents “cannot be detected”

Tumor tissue for genetic analyses was evaluated by frozen sections, and neoplastic cells accounted for 30–80% of viable cells. The total tumor RNA was extracted using the RNeasy Mini kit (Qiagen, Hilden, Germany), according to the manufacturers’ protocols. The concentrations of DNA and RNA were assessed using an absorption spectrometer. NCR-EW2, WES, and NCR-EW3 are Ewing sarcoma cell lines, and express *EWSR1-FLI1* (fusion variant 8 in Fig. 1), *EWSR1-ERG* (fusion variant 19 in Fig. 1) and *EWSR1-ETV4* (fusion variant 22 in Fig. 1), respectively [23]. NRS-1 (rhabdomyosarcoma cell line) and HEK293 total RNA were used as negative controls [24]. Total RNA was extracted from cells using ISOGEN (NIPPON GENE CO., LTD., Tokyo, Japan). The entire coding sequences of the *EWSR1-ETV1*, *EWSR1-FEV*, *FUS-ERG*, and *FUS-FEV* were constructed and subcloned into the pGEM-T vector (Promega, Madison, WI). The exon combinations of the control plasmids were *EWSR1* (NM_001163285.2) exon 7 – *ETV1* (NM_001163148.1) exon 11 (fusion variant 21 in Fig. 1), *EWSR1* exon 10 – *FEV* (NM_017521.2) exon 2 (fusion variant 23 in Fig. 1), *FUS* (NM_004960.3) exon 7 - *ERG* exon 11 (fusion variant 25 in Fig. 1), and *FUS* exon 10 – *FEV* exon 2 (fusion variant 28 in Fig. 1), respectively. Reverse transcription was performed using the Transcriptor First Strand cDNA Synthesis Kit with Oligo dT primers and random hexamers (Roche Diagnostics, Mannheim, Germany). The reaction temperature and time were applied in accordance with the protocol for long length mRNA recommended by the manufacturer’s protocol. Multiplex RT–PCR was performed using the Qiagen Multiplex PCR Plus Kit (Qiagen, Hilden, Germany). Conventional RT–PCR was performed using the QIAGEN HotStarTaq Plus Master Mix Kit (Qiagen, Hilden, Germany). Two sets of primers for multiplex RT–PCR were designed to detect fusion transcript variants. The primers are shown in Table 1. β-Actin primers used as the control were described elsewhere [25]. NCBI Primer-BLAST (https://www.ncbi.nlm.nih.gov/tools/primer-blast/) was used to design the primers, and the search conditions were set, so that the Tm of the primers was within 60 ± 3 °C, the maximum Tm difference was within 3 °C and an amplicon of the longest variant was within 1000 bp for Set A. As the forward primer of Set B, the most suitable primer was selected in combination with all reverse primers. It was confirmed by the Multiplex Primer Analyzer (Thermo Fisher SCIENTIFIC) that dimer formation did not theoretically occur in multiplex method. The optimal conditions for multiplex RT–PCR were as follows (based on the recommendation by the manufacturer’s protocol): final concentration of each primer is 0.2 μM, initial PCR activation at 95 °C for 5 min, 30–40 cycles of PCR consisting of denaturation at 95 °C for 30 s, annealing at 60 °C for 90 s and extension at 72 °C for 90 s, and final extension at 68 °C for 10 min. RT–PCR products of Set A and Set B were detected by electrophoresis using 2 and 1% agarose gel/ 1 × TAE buffer, respectively.

**Table 1:** Primer sequences for multiplex RT–PCR

Primer name		5′ ----- 3’	RT-PCR		Sequencing		
			Set A	Set B	F mix (set A)	F mix (set B)	R mix
EWSR1ex7_F (AF1)		gaacacctatgggcaaccga	✔		✔		
EWSR1ex4_F (BF1)		agaccgcctatgcaacttct		✔		✔	
FLI1ex9_R (R1)		ctcatcggggtccgtcattt	✔	✔			✔
ERGex12_R (R2)		cgtcatcttgaactccccgt	✔	✔			✔
ETV1ex11_R (R3)		atcctcgccgttggtatgtg	✔	✔			✔
ETV4ex11_R (R4)		gaccccttcctgcttgatgt	✔	✔			✔
FEVex2/3_R (R5)		gatctgtccgctgcctttct	✔	✔			✔
FUSex5_F (AF2)		ggacagcagaaccagtacaaca	✔		✔		
FUSex3_F (BF2)		cggacagcagagttacagtgg		✔		✔	

### Genomic PCR

The tumor genomic DNA was extracted using the QIAamp DNA Mini kit (Qiagen, Hilden, Germany), according to the manufacturers’ protocol. Genomic PCR was performed using AccuPrime Taq DNA Polymerase, high-fidelity (Invitrogen, Carlsbad, CA). The primers are listed in Supplementary Table S2.

### Sequencing analyses

Sequencing analysis was performed using ABI3130xl and ABI3500 (Applied Biosystems, Foster City, CA). The sequencing of the multiplex RT–PCR product was also performed using multiplex primers with 3.2 p mol each primer.

## Results

### Generation of the multiplex RT–PCR method to detect EWSR1/FUS-ETS

#### Design of the multiplex RT–PCR primers

We aimed to set up a multiplex RT-PCR system to detect all *EWSR1/FUS-ETS* fusion variants for rapid and practical genetic diagnosis. However, the exon combinations of the fusion gene are quite broad, and at least 28 types of variants have been reported (Fig. 1) [3, 5, 7–10, 23, 26–28]. There are 14 reported exon combinations of *EWSR1-FLI1*, all of which include exon 7 of *EWSR1*. Six combinations were reported in *EWSR1-ERG*. Five of them contained the complete exon 7 of *EWSR1*, whereas a single case only partially contained exon 7 [27]. One type of each was reported for *EWSR1-ETV1* and *EWSR1-ETV4*. Two types were reported for *EWSR1-FEV*. In summary, *EWSR1-ETS* contained the entire exon 7 of *EWSR1* except for one case. Therefore, a forward primer was designed to bind exon 7 of *EWSR1* as primer Set A. Reverse primers were designed to bind a common region in each fusion gene; that is, an *FLI1* primer was designed for exon 9, an *ERG* primer for exon 12, an *ETV1* primer for exon 11, and an *ETV4* primer for exon 11. The *FEV* primer spanned exon 2 to exon 3. Among *FUS-ERG* and *FUS-FEV* variants, the variant that fuses *FUS* exon 5 to *ERG* exon 9 has the shortest 5′-terminal side sequences of *FUS*. Therefore, a forward primer was designed in exon 5. Because all *FUS-ERG* variants include exon 12 of *ERG*, and *FUS-FEV* variants include exon 2 of *FEV*, the same reverse primers for *EWSR1-ETS* were used. The deduced size of each PCR product was 221 to 940 base pairs.

Next, we generated primer Set B that could detect fusion with a shorter 5′-terminal side sequence. The *EWSR1* forward primer was designed within exon 4 to detect the fusion gene that partially lacks exon 7. The *FUS* forward primer was designed within exon 3 to detect unusually short fusion genes, although no fusion with a shorter *FUS* 5′-terminal side sequence were reported in Ewing sarcoma. These forward primers were designed to match the reverse primer of Set A. Set A primers detect most variants, and the Set B primers cover all variants reported thus far (Table 1).

#### Sensitivity for the detection of EWSR1/FUS-ETS

**Fig. 2:**
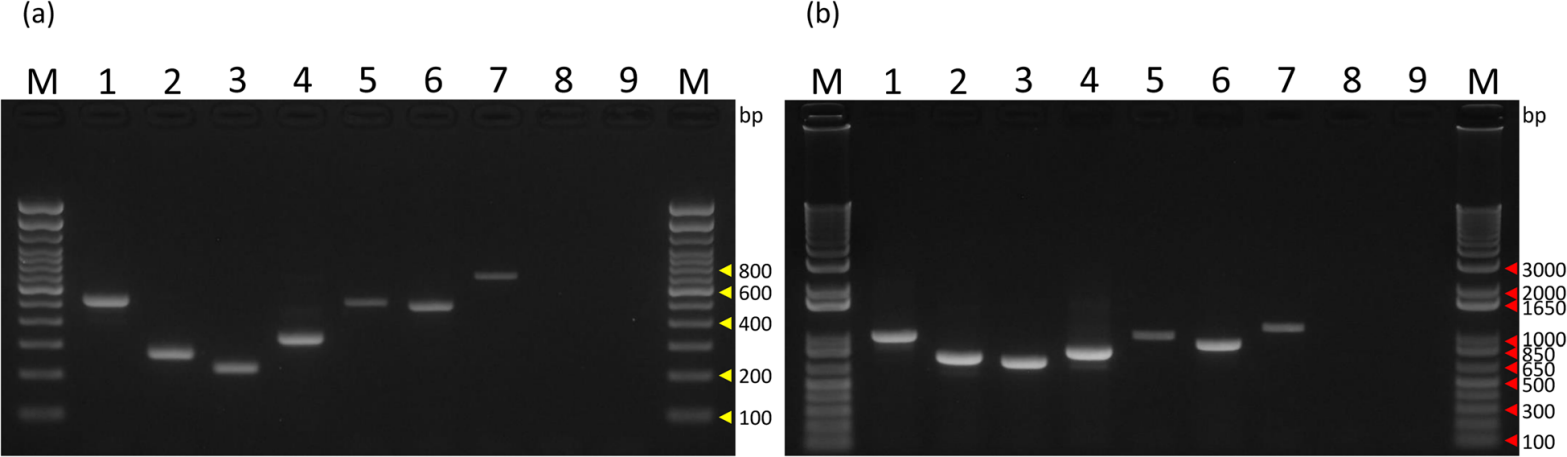
Specificity of multiplex primers to each fusion gene. Multiplex PCR for *EWSR1/FUS-ETS* transcript variants using Set A (a) and Set B (b) primers. Lane M: Trackit 100-bp ladder marker (a) or Trackit 1-kbp plus ladder (b). Molecular sizes are indicated in the right with yellow arrowheads (a) and red arrowheads (b), respectively.; lane 1: NCR-EW2 cDNA (*EWSR1-FLI1*); lane 2: WES cDNA (*EWSR1-ERG*); lane 3: *EWSR1-ETV1* plasmid; lane 4: NCR-EW3 cDNA (*EWSR1-ETV4*); lane 5: *EWSR1-FEV* plasmid; lane 6: *FUS-ERG* plasmid; lane 7: *FUS-FEV* plasmid; lane 8: HEK293 cDNA; lane 9: no template control. The plasmid samples contained the same amount of HEK293 cDNA as the Ewing cell lines

First, we performed PCR using primer Set A and cDNA from cell lines expressing either *EWSR1-FLI1* (NCR-EW2), *EWSR1-ERG* (WES), or *EWSR1-ETV4* (NCR-EW3) and diluted plasmid vectors (10^4^ molecules) containing *EWSR1-ETV1*, *EWSR1-FEV*, *FUS-ERG*, and *FUS-FEV* (Fig. 2a). A PCR product with expected length was identified in each reaction without recognizable background. Similarly, fusion genes with the expected length were amplified by PCR using Set B primers (Fig. 2b). In all cases, only a single band was detected with low background. All PCR products amplified with either Set A or Set B were sequenced successfully using the forward or reverse primer mix. Sequence analysis showed that all PCR products were the expected sequences of the *EWSR1/FUS-ETS* variants.

Next, we examined the sensitivity of the PCR. We performed RT–PCR using Set A primers and cDNA from cell lines expressing either *EWSR1-FLI1*, *EWSR1-ERG*, or *EWSR1-ETV4*. We detected the respective fusion genes from cDNA equivalent to 100 pg of RNA using 35 cycles, according to the cycle number of the existing analysis (Supplementary Fig. S1). RT–PCR using Set B primers had comparable sensitivity with RT–PCR using Set A primers (Supplementary Fig. S1). For fusion transcripts without cell lines, we used a dilution series of plasmid vectors. Using Set A primers, positive results were obtained with 10^2^ plasmid molecules for *EWSR1-ETV1* and *FUS-ERG*, and 10^3^ molecules for *EWSR1-FEV* and FUS-FEV (Supplementary Fig. S2). The sensitivity of Set B was comparable with that of Set A. Similarly, we performed the PCR at 40 cycles, and obtained a clear band with less template (data not shown).

#### Detection of EWSR1/FUS-ETS in clinical samples

**Fig. 3:**
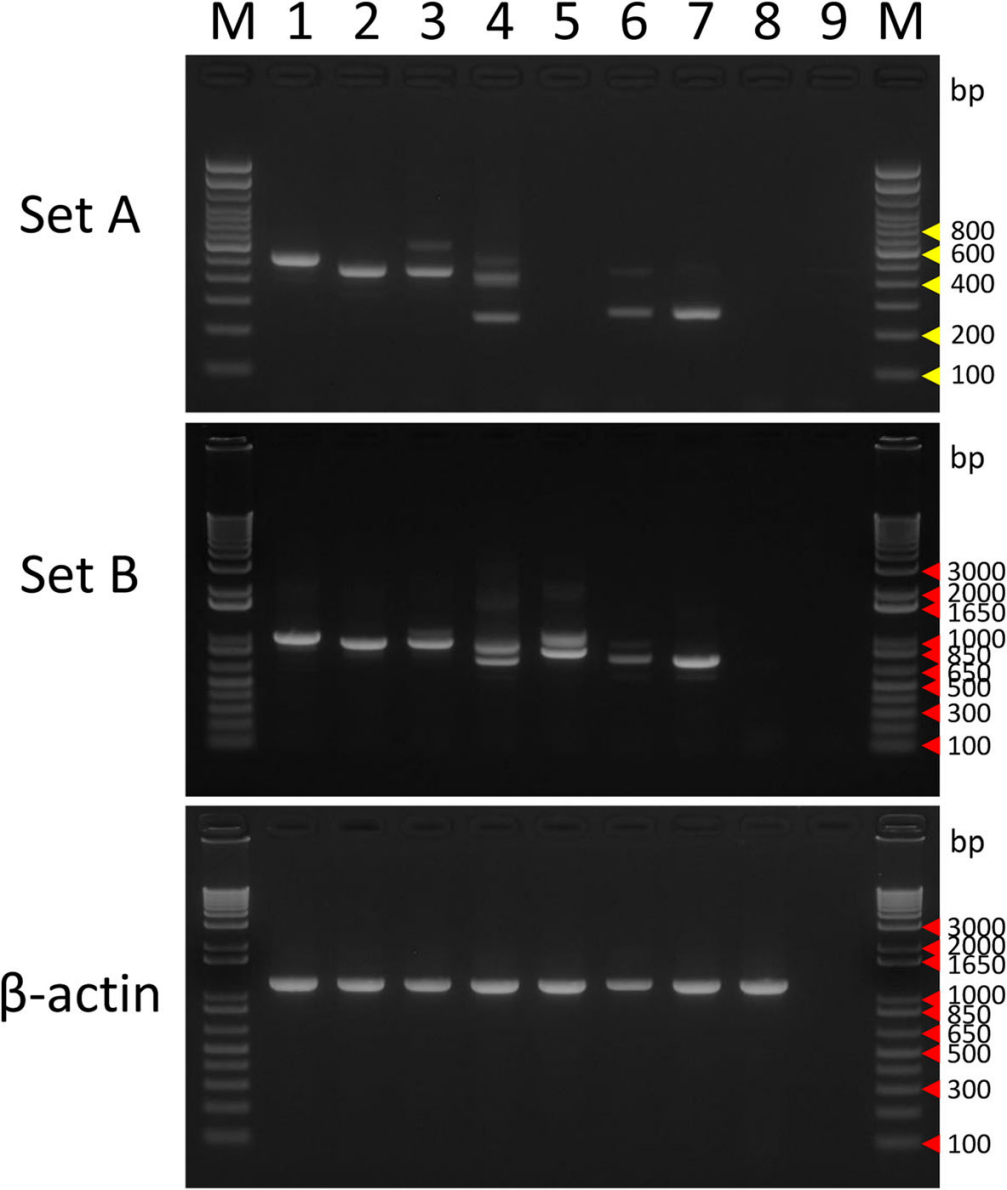
Multiplex RT–PCR in tumor samples. Multiplex (top and middle panels) and control (bottom panel) RT–PCR were performed in six tumor samples. Lane M: Trackit 100-bp ladder marker (top panel) or Trackit 1-kbp plus ladder (middle and bottom panels). Molecular sizes are indicated in the right with yellow arrowheads (a) and red arrowheads (b), respectively.; lane 1: NCR-EW2 cDNA for the positive control; lane 2: tumor 1; lane 3: tumor 2; lane 4: tumor 3; lane 5: tumor 4; lane 6: tumor 5; lane 7: tumor 6; lane 8: NRS-1 cDNA for the negative control; lane 9: no template control

We examined the usefulness of these primer sets using clinical samples. We utilized frozen material from Ewing sarcoma diagnosed morphologically, immunohistochemically, and genetically. Specifically, six small round cell tumors with membranous CD99-positivity and *EWSR1* rearrangement by FISH or RT–PCR were analyzed. The fusion gene of tumor 4 was not detected by existing RT–PCR. Using Set A, we detected *EWSR1/FUS-ETS* in five of six cases (Fig. 3). Sequence analysis confirmed that tumors 1 and 2 had *EWSR1-FLI1*, tumor 3 had *EWSR1-FEV*, and tumors 5 and 6 had *EWSR1-ERG* (Table 2). Using PCR with Set B, we identified *EWSR1/FUS-ETS* in all six cases (Fig. 3). We detected three bands in tumor 4 using Set B primers. Sequence analysis revealed that these bands were *EWSR1-ERG*, but the fusion point was unclear because of multiple PCR products. Accordingly, we examined this fusion gene in detail and reviewed the clinicopathological features of this case.

**Table 2:** Fusion transcripts identified in tumor samples

Tumor	Fusion Transcripts^a^
1	*EWSR1* exon 7 - *FLI1* exon 6 (fusion variant 11)
2	*EWSR1* exon 7 - *FLI1* exon 6 (fusion variant 11)
3	*EWSR1* exon 7 - *FEV* exon 2 (fusion variant 24), *EWSR1*⊿exon 8 (c.794 to 943)-*FEV* exon 2
4	*EWSR1* ⊿exon 6 (c.414 to 522) - intron 6 - intron 6 / *ERG* intron 8 - exon 9
5	*EWSR1* exon 7 - *ERG* exon 12 (fusion variant 19)
6	*EWSR1* exon 7 - *ERG* exon 12 (fusion variant 19)

^a^Only the in-frame fusions are described here. The fusion variant numbers in Fig. 1 are shown in parentheses.

### A unique case of an EWSR1-ERG-expressing tumor

#### Clinical and pathological characteristics

**Fig. 4:**
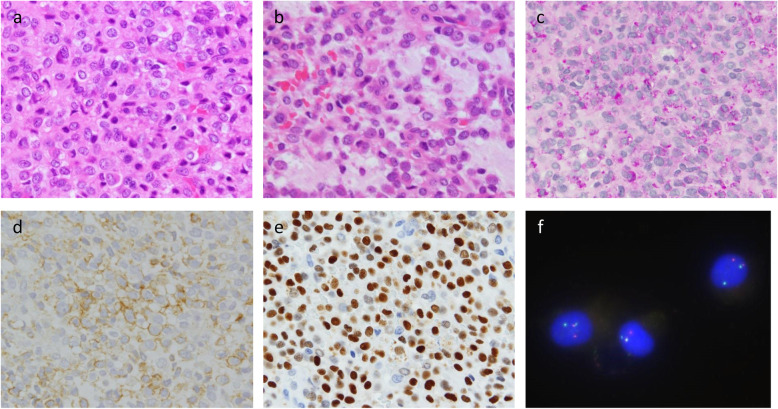
Pathological features of the tumor and FISH analysis. The pathological features of tumor 4 are shown. **a**, **b** Hematoxylin and eosin staining. **c** PAS staining. **d** Immunohistochemistry of CD99. **e** Immunohistochemistry of Nkx2.2. (f) FISH analysis using the *EWSR1* break-apart probe

We reviewed the clinical and pathological characteristics. The patient was a 15-year-old male with a history of acute lymphoblastic leukemia who presented with a mass measuring 1.5 × 1.0 × 0.7 cm in his nasal vestibule. The tumor was subjected to excisional biopsy. Histologically, the tumor exhibited diffuse proliferation of undifferentiated cells (Fig. 4a) with round to oval nuclei and a moderate amount of cytoplasm with a clear cell border. Focally, the tumor cells proliferated with fibrous to myxoid stroma (Fig. 4b) and were positive for Periodic acid–Schiff (PAS) staining in the cytoplasm (Fig. 4c). Immunohistochemically, tumor cells demonstrated membranous positivity for CD99, positivity for Nkx2.2 (Fig. 4d and e), focal positivity for S100 and negativity for desmin, myogenin, MyoD1, cytokeratin (AE1/AE3), CD31, CD34, CD3, CD20, and CD1a. FISH analysis of fresh-tissue touch preparations detected *EWSR1* split signals in most tumor cells but not *FUS* split signals (Fig. 4f). The tumor had consistent features of Ewing sarcoma based on CD99 positivity and *EWSR1* rearrangement, although the histological picture was somewhat unusual in that it showed focal myxoid stroma.

#### Detailed analysis of the fusion transcript

**Fig. 5:**
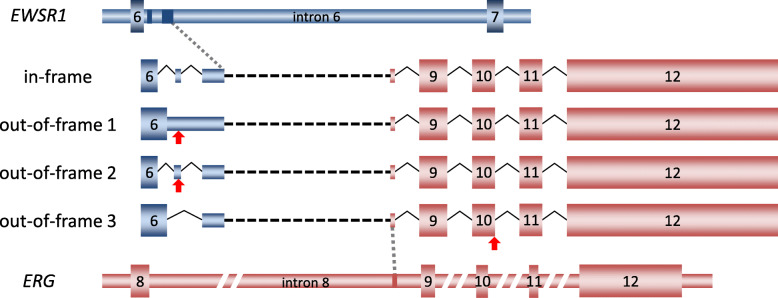
Schematic representations of the fusion transcripts in tumor 4. Scheme of the transcript variants of *EWSR1-ERG*. The blue boxes in intron 6 of *EWSR1* and red box in intron 8 of *ERG* indicate cryptic exons found in the in-frame variant. The red arrows in out-of-frame variants are sites of the termination codon. EWSR1 and ERG are connected by black dotted lines

Sequence analysis of the multiplex PCR product revealed that the fusion gene was *EWSR1-ERG*. To determine the sequence of the individual products, we performed another PCR assay using *EWSR1* exon 4 and *ERG* exon 12 primers, and the products were subcloned into the pGEM-T vector and sequenced. We identified four transcript variants, and the most frequent one was in-frame (Fig. 5). The major in-frame *EWSR1-ERG* fusion transcript variant included a partial sequence of exon 6 (c.414 to c.522), two cryptic exons (c.581 + 55 to + 90, c.581 + 227 to + 369) in intron 6 of *EWSR1*, a cryptic exon in intron 8 of *ERG* (c.767–214 to − 198), and exon 9 of *ERG* (Fig. 5 and Supplementary Fig. S3a, b and c). All four variants had identical sequences from *ERG*. Three out-of-frame variants were thought to be produced by differential splicing within *EWSR1* (Fig. 5 and Supplementary Fig. S3a, b and c).

#### Genomic structure of the EWSR1-ERG fusion

**Fig. 6:**
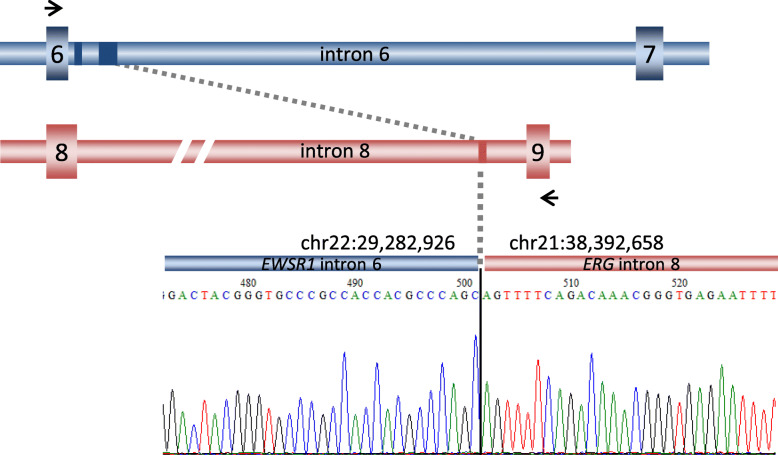
Genomic fusion point of novel *EWSR1-ERG* translocation. Sequences of the genomic fusion point. The blue boxes indicate cryptic exons in the in-frame transcript. The black arrows indicate primer sites. In the upper part of the sequences, the chromosome positions of the fusion site are indicated

To clarify whether the rare variants were derived from alternative splicing or different breakpoints in genomic DNA, we performed genomic PCR and identified a single fused sequence showing that intron 6 of *EWSR1* was joined to intron 8 of *ERG* (Fig. 6). The genomic fusion point was identical to the fusion points in transcripts, indicating a part of *EWSR1* intron 6 and a short sequence of *ERG* intron 8 formed a cryptic exon collectively. Notably, all flanking sequences of all cryptic exons followed the GU/AG mRNA splicing rule, and all four transcript variants were supposed to be derived from alternative splicing.

## Discussion

We developed a novel RT–PCR assay that can efficiently detect both *EWSR1-ETS* and *FUS-ETS* observed in Ewing sarcoma. We identified *EWSR1/FUS-ETS* fusion transcripts in all cell lines and pathologically defined Ewing sarcoma tumors that were tested. The appropriately designed primers enabled the detection of various fusion variants in a single round of PCR. Additionally, the identified transcripts were successfully sequenced by mixed forward or reverse primers in each case. Among 28 EWS/FUS-ETS variants reported so far, we detected five using cell lines and tumor tissues, and additionally, we identified a novel variant.

We were able to detect the fusion genes from 100 pg of total RNA from cell lines. In addition, we detected 1000 molecules of the fusion gene in a PCR reaction. For clinical samples, we use 1/40 of the cDNA synthesized with 1 μg of total RNA as the PCR template. Assuming that the amount of total RNA per cell is 0.01 ng, the template used for PCR is theoretically equivalent to 2500 cells. Thus, although the number of samples analyzed was small, our method is theoretically applicable to clinical tumor samples.

In 1995, Downing et al. reported multiplex RT-PCR for the detection of *EWSR1-FLI1* and *PAX3-FOXO1* to differentiate Ewing sarcoma and alveolar rhabdomyosarcoma [19]. However, many fusion variants were discovered afterwards. In addition, the primers they used cannot detect fusion variants with a short N-terminal sequence. In 2001, Peter et al. used a real-time PCR system to discriminate Ewing sarcoma, alveolar rhabdomyosarcoma, synovial sarcoma, and small round cell desmoplastic tumor [20]. They used primers only for *EWSR1-ETS*. As they utilized a common *EWSR1* probe for the detection of the amplicon, they were unable to differentiate fusion gene combinations. Moreover, the product sizes were too large for the real-time PCR method. Yoshino et al. reported the simultaneous detection of *EWSR1-ETS* in 2003 [21]. They used Bioanalyzer to confirm the product length, which made it possible to predict the gene and exon combinations, but they utilized *EWSR1* exon 7 primer and were unable to detect fusion with a short N-terminal sequence. Thus, none of these assays were able to detect *FUS-ETS* fusions. In routine pathological practice, Ewing sarcoma with atypical morphology or small round cell sarcoma without typical *EWSR1/FUS-ETS* fusions is occasionally observed. Therefore, rapid detection of all Ewing sarcoma related fusions is useful in clinical settings. The advantages of conventional RT–PCR with gel electrophoresis are that it is inexpensive and not laborious, and the length of the product can be recognized. In addition, all the processes can be performed without special equipment such as a real-time PCR system or next-generation sequencer. The standard break-apart FISH method takes 2 days to determine EWSR1 and FUS rearrangement [29], and it takes another 2 days to determine the fusion partner. However, our method enables confirmation of the presence of the EWSR1/FUS-ETS fusion gene in 5.5 h and determination of the sequence in approximately 10 h.

Furthermore, we reported a case of Ewing sarcoma with atypical histological features whose fusion transcript completely lacked exon 7 of *EWSR1*. By our methods, the use of the *EWSR1* upstream forward primer enabled the detection of a transcript variant with an unexpectedly shorter N-terminal region of *EWSR1*. Many laboratories detect fusion genes by RT–PCR, but most primers reported thus far cannot amplify fusion transcripts lacking *EWSR1* exon 7. Thus, false-negative results may occur with *EWSR1-ETS* and *EWSR1-ERG*. Therefore, when Ewing sarcoma is pathologically suspected, but all seven types of known fusion genes are negative, analysis using an EWSR1 primer upstream of exon 7 should be considered.

## Conclusions

We developed a multiplex PCR assay method that is simple, accurate, and efficient to detect fusion genes observed in Ewing sarcoma. Our assay will aid in the rapid and accurate diagnosis of Ewing sarcoma. We also identified a novel fusion variant with a short N-terminal region that may have been previously overlooked. This highlights why the RT–PCR primers for the genetic diagnosis of Ewing sarcoma should be optimized.

## Supplementary Information

**Additional file 1: Supplementary Table S1.** List of antibodies.

**Additional file 2: Supplementary Table S2**. Sequences of genomic PCR and sequencing primers.

**Additional file 3: Supplementary Fig. S1.** Detection sensitivity of primers for *EWSR1-ETS*cDNA. Serial dilutions of cDNA from Ewing sarcoma cell lines were amplified by Set A (upper panel) or Set B primers (lower panel). Lane M: Trackit100-bp ladder marker (upper panel, yellow arrowhead) or Trackit1-kbp plus ladder (lower panel, red arrowhead), molecular marker sizes are indicated intheleft.; lane 1: template cDNA corresponding to 10 ng to total RNA; lane 2: 1 ng; lane 3: 100 pg; lane 4: 10 pg; lane 5: 1 pg; lane 6: 0.1 pg; lane 7: no template control.

**Additional file 4: Supplementary Fig. S2.** Detection sensitivity of primers for *EWSR1-ETS*plasmids. Serial dilutions of *EWSR1/FUS-ETS*-containing plasmids were amplified using primer Set A (upper panels) and Set B (lower panels).We estimated the molecular weight from the size of each plasmid, andmade serial dilution of the respective plasmids and used 105to 100molecules as a starting template in 25 μlof the PCR reaction mix. Lane M: Trackit100-bp ladder marker (upper panel, yellow arrowhead) or Trackit1-kbp plus ladder (lower panel, red arrowhead), molecular marker sizes are indicated in the left.; lane 1: 105molecules; lane 2: 104molecules; lane 3: 103molecules; lane 4: 102molecules; lane 5: 101molecules; lane 6: 100molecule; lane 7: no template control.

**Additional file 5: Supplementary Fig. S3.** Scheme of alternative splicing and sequence in novel *EWSR1-ERG*transcripts. (a) Scheme of exon 6 and first 369 bases of intron 6 of *EWSR1*. The splicing patterns of the in-frame variant are represented by red lines and a red number. The splicing sites shared by some variants are denoted by black numbers. The two blue bars under intron 6 are cryptic exonicregions of the in-frame variant. The splicing patterns of the two out-of-frame variants are shown in purple and green, respectively. No sequence was spliced out in one variant. (b) Sequences of *EWSR1*exon 6 and intron 6 (c.581 + 1 to + 393) and alternative splicing sites. The genomic location numbers are based on the GRCh37/hg19 version. The boldfaced sequences represent truncated exon 6 (c.414 to c.522). The sequences corresponding to the cryptic exon in intron 6 are underlined. GT/gt(green character): splice donor sites, ag (red character): splice acceptor sites. Sites involved in splicing are numbered in the upper part of the sequences. (c) Sequences of *ERG* intron 8 (c.767–1 to − 230) and exon 9 and alternative splicing sites. The genomic location numbers and sites involved in splicing are shown as in (b).

## Data Availability

Not applicable.

## Abbreviations

RT-PCR: Reverse transcription–polymerase chain reaction
FISH: Fluorescence in situ hybridization
PAS: Periodic acid–Schiff
