# Supplementary material for: Establishment of multiplex RT-PCR to detect fusion genes for the diagnosis of Ewing sarcoma
Source: Diagn Pathol. 2021 Nov 8;16:102. doi: 10.1186/s13000-021-01164-6 (PMC8573982; doi:10.1186/s13000-021-01164-6)
Supplement: Supplementary file 1 — Additional file 1: Supplementary Table S1. List of antibodies. [file 13000_2021_1164_MOESM1_ESM.pdf]

**Supplementary Table S1.** List of antibodies

| Antibody | Clone | Dilution | Resource |
|---|---|---|---|
| CD99 | O13 | 1:800 | ThermoFischer Scientific |
| Nkx2.2 | 74.5A5 | 1:200 | BD Biosciences |
| S100 | - | 1:2000 | DakoCytomation (agilent) |
| desmin | D33 | 1:100 | DakoCytomation (agilent) |
| myogenin | F5D | 1:100 | DakoCytomation (agilent) |
| Myo-D1 | 5.8A | 1:50 | DakoCytomation (agilent) |
| cytokeratin | AE1/AE3 | 1:1 | NICHIREI BIOSCIENCES |
| CD31 | JC70A | 1:100 | DakoCytomation (agilent) |
| CD34 | QBEnd10 | 1:100 | DakoCytomation (agilent) |
| CD3 | PS1 | 1:1 | NICHIREI BIOSCIENCES |
| CD20 | L26 | 1:200 | DakoCytomation (agilent) |
| CD1a | O10 | 1:2 | MBL |
