# Supplementary material for: Establishment of multiplex RT-PCR to detect fusion genes for the diagnosis of Ewing sarcoma
Source: Diagn Pathol. 2021 Nov 8;16:102. doi: 10.1186/s13000-021-01164-6 (PMC8573982; doi:10.1186/s13000-021-01164-6)
Supplement: Supplementary file 2 — Additional file 2: Supplementary Table S2. Sequences of genomic PCR and sequencing primers. [file 13000_2021_1164_MOESM2_ESM.pdf]

Supplementary Table S2. Sequences of genomic PCR and sequencing primers

**Primer name**	**Sequence (5’ --- 3’)**
EWSR1_fw28	cctcaatctagcacaggggg
ERG_ex9/int9_rv	agccaacactgtacctttcgac
