# Supplementary material for: Establishment of multiplex RT-PCR to detect fusion genes for the diagnosis of Ewing sarcoma
Source: Diagn Pathol. 2021 Nov 8;16:102. doi: 10.1186/s13000-021-01164-6 (PMC8573982; doi:10.1186/s13000-021-01164-6)
Supplement: Supplementary file 3 — Additional file 3: Supplementary Fig. S1. Detection sensitivity of primers for EWSR1-ETScDNA. Serial dilutions of cDNA from Ewing sarcoma cell lines were amplified by Set A (upper panel) or Set B primers (lower panel). Lane M: Trackit100-bp ladder marker (upper panel, yellow arrowhead) or Trackit1-kbp plus ladder (lower panel, red arrowhead), molecular marker sizes are indicated intheleft.; lane 1: template cDNA corresponding to 10 ng to total RNA; lane 2: 1 ng; lane 3: 100 pg; lane 4: 10 pg; lane 5: 1 pg; lane 6: 0.1 pg; lane 7: no template control. [file 13000_2021_1164_MOESM3_ESM.pdf]

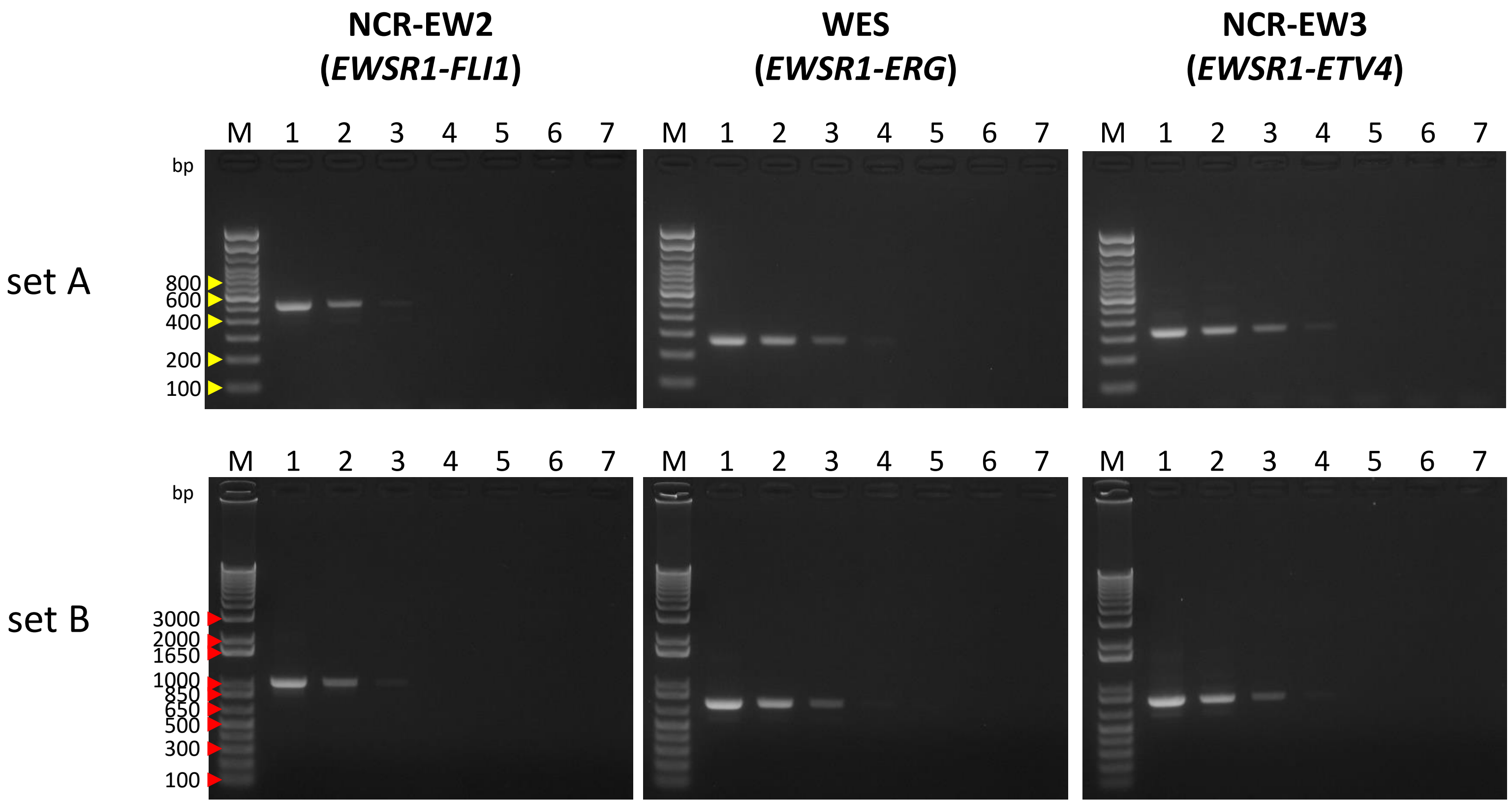

Supplementary Figure S1. Detection sensitivity of primers for *EWSR1-ETS* cDNA

Serial dilutions of cDNA from Ewing sarcoma cell lines were amplified by Set A (upper panel) or Set B primers (lower panel). Lane M: Trackit 100-bp ladder marker (upper panel, yellow arrowhead) or Trackit 1-kbp plus ladder (lower panel, red arrowhead), molecular marker sizes are indicated in the left.; lane 1: template cDNA corresponding to 10 ng to total RNA; lane 2: 1 ng; lane 3: 100 pg; lane 4: 10 pg; lane 5: 1 pg; lane 6: 0.1 pg; lane 7: no template control.
