# Supplementary material for: Establishment of multiplex RT-PCR to detect fusion genes for the diagnosis of Ewing sarcoma
Source: Diagn Pathol. 2021 Nov 8;16:102. doi: 10.1186/s13000-021-01164-6 (PMC8573982; doi:10.1186/s13000-021-01164-6)
Supplement: Supplementary file 4 — Additional file 4: Supplementary Fig. S2. Detection sensitivity of primers for EWSR1-ETSplasmids. Serial dilutions of EWSR1/FUS-ETS-containing plasmids were amplified using primer Set A (upper panels) and Set B (lower panels).We estimated the molecular weight from the size of each plasmid, andmade serial dilution of the respective plasmids and used 105to 100molecules as a starting template in 25 μlof the PCR reaction mix. Lane M: Trackit100-bp ladder marker (upper panel, yellow arrowhead) or Trackit1-kbp plus ladder (lower panel, red arrowhead), molecular marker sizes are indicated in the left.; lane 1: 105molecules; lane 2: 104molecules; lane 3: 103molecules; lane 4: 102molecules; lane 5: 101molecules; lane 6: 100molecule; lane 7: no template control. [file 13000_2021_1164_MOESM4_ESM.pdf]

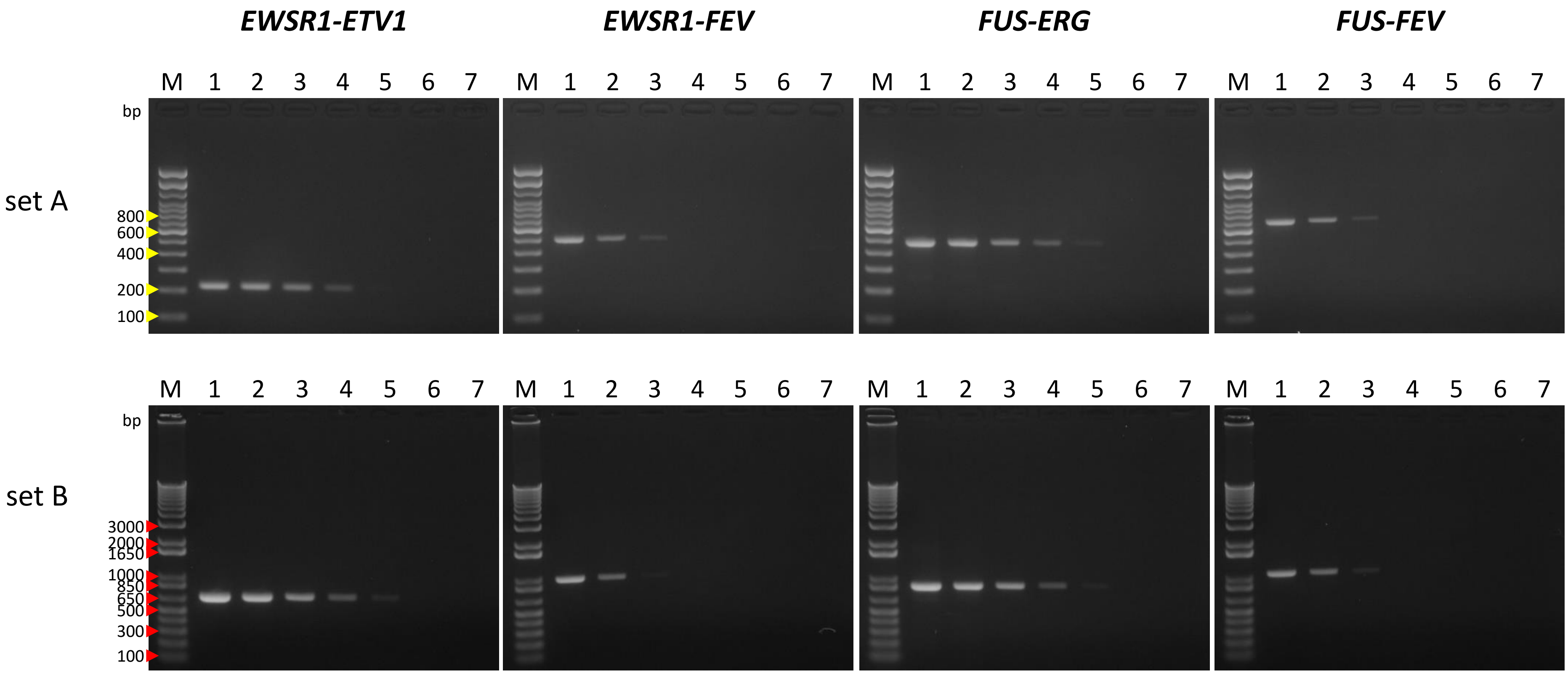

Supplementary Figure S2. Detection sensitivity of primers for *EWSR1-ETS* plasmids

Serial dilutions of *EWSR1/FUS-ETS*-containing plasmids were amplified using primer Set A (upper panels) and Set B (lower panels). We estimated the molecular weight from the size of each plasmid, and made serial dilution of the respective plasmids and used 10^5 to 10^0 molecules as a starting template in 25 μl of the PCR reaction mix. Lane M: Trackit 100-bp ladder marker (upper panel, yellow arrowhead) or Trackit 1-kbp plus ladder (lower panel, red arrowhead), molecular marker sizes are indicated in the left. ; lane 1: 10^5 molecules; lane 2: 10^4 molecules; lane 3: 10^3 molecules; lane 4: 10^2 molecules; lane 5: 10^1 molecules; lane 6: 10^0 molecule; lane 7: no template control.
