# Supplementary material for: Establishment of multiplex RT-PCR to detect fusion genes for the diagnosis of Ewing sarcoma
Source: Diagn Pathol. 2021 Nov 8;16:102. doi: 10.1186/s13000-021-01164-6 (PMC8573982; doi:10.1186/s13000-021-01164-6)
Supplement: Supplementary file 5 — Additional file 5: Supplementary Fig. S3. Scheme of alternative splicing and sequence in novel EWSR1-ERGtranscripts. (a) Scheme of exon 6 and first 369 bases of intron 6 of EWSR1. The splicing patterns of the in-frame variant are represented by red lines and a red number. The splicing sites shared by some variants are denoted by black numbers. The two blue bars under intron 6 are cryptic exonicregions of the in-frame variant. The splicing patterns of the two out-of-frame variants are shown in purple and green, respectively. No sequence was spliced out in one variant. (b) Sequences of EWSR1exon 6 and intron 6 (c.581 + 1 to + 393) and alternative splicing sites. The genomic location numbers are based on the GRCh37/hg19 version. The boldfaced sequences represent truncated exon 6 (c.414 to c.522). The sequences corresponding to the cryptic exon in intron 6 are underlined. GT/gt(green character): splice donor sites, ag (red character): splice acceptor sites. Sites involved in splicing are numbered in the upper part of the sequences. (c) Sequences of ERG intron 8 (c.767–1 to − 230) and exon 9 and alternative splicing sites. The genomic location numbers and sites involved in splicing are shown as in (b). [file 13000_2021_1164_MOESM5_ESM.pdf]

Supplementary Figure S3

a

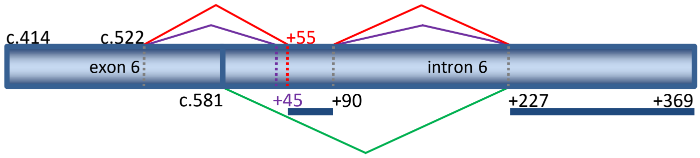

b

EWSR1 exon 6 (chr22: 29,282,390 - 29,282,557,strand= +, c.414-c.581)

ACCGCAGGATGGAAACAAGCCCACTGAGACTAGTCAACCTCAATCTAGCA
CAGGGGGTTACAACCAGCCCAGCCTAGGATATGGACAGAGTAACTACAGT
TATCCCCAGGTACCTGGGAGCTACCCCATGCAGCCAGTCACTGCACCTCC
ATCCTACCCTCCTACCAG

EWSR1 intron 6 (chr22: 29,282,558 - 29,282,950, stand=+)

+1 +45
gtcagtctactttttgtggcaaaacaaaaacagtgacaaaacagtttttt

+55 +90
tcaggttgttgaccagcttgctttgacttcttcagctaaggtatgttatc

tgactgtcgcagtgataagtcatctgaccatacttggttacattaaagcc

tgagatctagggtcccatagtgttgcctggcagctaataactattctttt

+227
ttctttttttttttttttcccccgagacggagtctctctctgtcgcccag

gctggagtgcagtggcgcgatctcggctcactgcaagctccacctcctgg
gttcacgccattctcctgcctcaacctcccgagtagctgggactacgggt

+369
gcccgccaccacgcccagctaatttttttgtatttttagtagag-----

c

ERG intron 8 (chr21: 38392674 - 38392445 strand= -)

-214 -198

----------agaatctgccaatggaagttttcagacaaacgggtgagaa
ttttagaccactataatatatgaaattttaatttgtaaataaaactcaaa
tctagtgggatatttctttggattaccagatttttttgcacaaagtttat
tctaataaatacaaaatcaaataaaaaaccaaagcatctcttataaaaat

-1

tacatgattgatcttttagtcaatttatttgtctttacag

ERG exon 9 (chr21: 38,392,376 - 38,392,444 strand= - , c.767-c.835)

ATTTACCATATGAGCCCCCCAGGAGATCAGCCTGGACCGGTCACGGCCAC
CCCACGCCCCAGTCGAAAG

Supplementary Figure S3. Scheme of alternative splicing and sequence in novel *EWSR1-ERG* transcripts

(a) Scheme of exon 6 and first 369 bases of intron 6 of *EWSR1*. The splicing patterns of the in-frame variant are represented by red lines and a red number. The splicing sites shared by some variants are denoted by black numbers. The two blue bars under intron 6 are cryptic exonic regions of the in-frame variant. The splicing patterns of the two out-of-frame variants are shown in purple and green, respectively. No sequence was spliced out in one variant. (b) Sequences of *EWSR1* exon 6 and intron 6 (c.581+1 to +393) and alternative splicing sites. The genomic location numbers are based on the GRCh37/hg19 version. The boldfaced sequences represent truncated exon 6 (c.414 to c.522). The sequences corresponding to the cryptic exon in intron 6 are underlined. GT/gt (green character): splice donor sites, ag (red character): splice acceptor sites. Sites involved in splicing are numbered in the upper part of the sequences. (c) Sequences of *ERG* intron 8 (c.767-1 to -230) and exon 9 and alternative splicing sites. The genomic location numbers and sites involved in splicing are shown as in (b).
